# Editorial “Embodiment in the Metaverse: How Real and Virtual Bodies in Interaction Affect Cognition”

**DOI:** 10.5334/joc.374

**Published:** 2024-06-18

**Authors:** Claudia Repetto, Giuseppe Riva

**Affiliations:** 1 Dept. of Psychology, UniversitàCattolica del Sacro Cuore, Mila, Italy; 2 Human Technology Lab, UniversitàCattolica del Sacro Cuore, Milan, Italy; 3 Applied Technology for Neuro-Psychology Lab, IRCCS Istituto Auxologico Italiano, Milan, Italy

When we move into the world, we are immediately aware of being the owners of our bodies and being able to fully control our actions (Repetto & Riva, 2023; Riva, 2018). The first is referred to as the sense of ownership, and the second as the sense of agency, and both are key determinants of the sense of Embodiment (Longo & Haggard, 2012), which plays a fundamental role in bodily self-consciousness (BSC).

**Figure 1:**
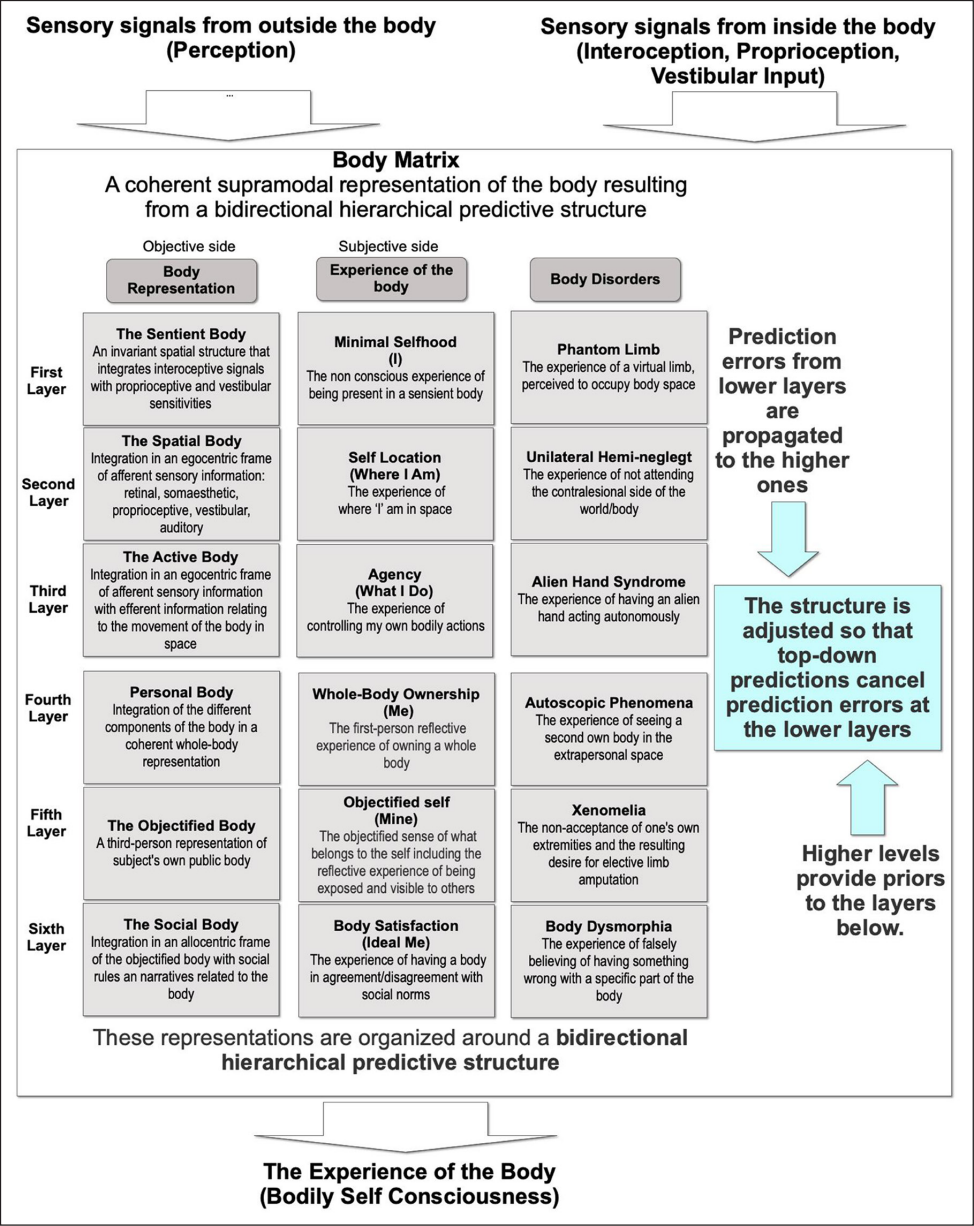
Bodily Self-Consciousness (adapted from Riva, 2018).

However, the study of BSC is not an easy task. Even if BSC is for the individual a unitary experience, neuroimaging and neurological data suggested that BSC includes different layers (Figure 1) that integrate both sensory and cognitive bodily data in a coherent experience.

For this reason, the study of BSC has centered on clinical populations exhibiting altered perceptions of their bodies and on healthy individuals subjected to experimental paradigms capable of disrupting multisensory integration. More recently, the advent of Immersive Virtual Reality (IVR) technology has ushered in a new era, allowing researchers to investigate embodiment in a completely novel way (Matamala-Gomez et al., 2021).

IVR shares a fundamental mechanism with the human brain—embodied simulations, which are central to the theory of predictive coding (Talsma, 2015). This concept in neuroscience suggests that the brain constructs and updates an internal model of the body and the environment around it to anticipate sensory inputs, rather than just reacting to it. IVR operates similarly by creating an avatar interacting in a computer-generated world that users can use and manipulate as if they were in (Riva et al., 2019). Specifically, IVR aims to mimic the sensory responses we expect from real-life actions, offering an avatar and a virtual environment that adjusts dynamically to the user’s actions. The more accurately the IVR mirrors the brain’s internal models, the more realistic and persuasive the virtual experience feels.

In this view, IVR provides unique opportunities to study the process of embodiment, as the user can “wear” a virtual body that, in turn, can be manipulated in ways that defy real-world constraints (Kilteni et al., 2012).

In the contemporary landscape, IVR stands poised to advance further with the emergence of the Metaverse. The Metaverse is a simulated digital environment that incorporates augmented reality (AR), virtual reality (VR), and Artificial Intelligence (AI), to build spaces where users can interact as in the actual world. In this way, we might think of the Metaverse as the next evolutionary step of the internet and the digital technologies, where the boundaries between digital and physical will blur (Riva & Wiederhold, 2022). Psychologists and neuroscientists can’t help but wonder how the sense of Embodiment may be altered in the Metaverse, where the real and virtual bodies could interact in intricate ways. For instance, I could make a real action and consistently see my virtual avatar performing the same action; or, rather, I could use my real hand to move my virtual leg, determining a discrepancy between the real and virtual effectors. More, being immersed in an environment populated by other avatars in interaction, with rich and real-like bodily cues (e.g. gestures, facial expressions), also the observation of others’ actions and bodies must be integrated with one’s own sense of Embodiment, opening up unprecedented research questions (Cerasa et al., 2024).

Furthermore, most of IVR research, including work from a technological perspective, has focused on how external bodily information (i.e., the avatar) is processed, integrated, and contributes to our sense of self. Despite the accomplishments of such efforts, what truly distinguishes our bodies from other physical objects (Figure 1) is that we do not solely perceive them through external senses (exteroception). We also have an internal mean of accessing our bodies through interoceptive signals (such as those related to our physiological state), proprioceptive signals (such as those related to our body positioning and movement), and vestibular signals (such as those related to our sense of balance). Excitingly, the emerging metaverse now allows us to modify these internal signals as well, through the development of interoceptive technologies (Schoeller et al., 2024). These technologies can generate artificial sensations that directly influence bodily signals (Di Lernia et al., 2018), create interoceptive illusions that modulate the context to influence interoception (Iodice et al, 2019), and facilitate systems of emotional augmentation (Schoeller et al., 2019). For example, Di Lernia and colleagues (Di Lernia et al., 2023) used synthetic auditory frequencies at 6 Hz and 2 Hz to activate respectively the left and right insula and, in turn, to enhance the cortical processing of interoceptive signals.

A scholarly examination of these themes is both timely and imperative, not only to understand how our body representation *per se* could change in the Metaverse, but more so considering the pivotal role of the body for cognitive processing. Decades of research have underscored the active involvement of the sensorimotor system in knowledge construction, memory encoding and retrieval, spatial processing, and language comprehension and production (Barsalou, 2008). If this olds true in conventional life settings, how might alterations in the sense of Embodiment impact cognitive processes within the Metaverse?

This Special collection endeavours to convene experts from cognitive psychology and neuroscience, to reflect on the challenges and opportunities presented by the Metaverse to the scientific community. In particular, by delving into the study of embodiment within the Metaverse, the collection aims to envision future scenarios for leveraging this technology in both healthy individuals and patients.

The Special Collection includes three papers offering multifaceted insights into the subject matter. Parsons (2024) emphasizes the role of the Metaverse in expanding our understanding of cognitive processes and ecological validity in research. He posits that the Metaverse has the potential to transcend the constraints of traditional laboratory settings and conventional brain-centric approaches and even the limitations of brain-based cognition. According to the author, the embodiment within Metaverse technologies, encompassing sensory-motor experiences that integrate various forms of bodily sensations, harmonizes these sensory inputs with the user’s multimodal neural networks. Metaverse technologies, such as virtual reality, leveraging artificial neural networks and machine learning algorithms can predict the sensory consequences of user actions, thereby immersing the user in a virtual environment where anticipated outcomes align with real-world neural responses. Overall, the Metaverse is presented as a transformative tool for advancing our understanding of cognition and expanding the concept of ecological validity in cognitive research.

In their paper, Pascucci and collaborators (Pascucci et al., 2024) investigate the virtual embodiment in the context of the Michelangelo effect, wherein painting a virtual art masterpiece yields less fatigue and motor errors than colouring control canvas. In this study the authors prompted the participants to virtually sculpt either some famous sculptures in the history of art or some control stimuli, using their real hands digitally reproduced in the virtual environment. They found that engaging with the experimental statues influenced the fluidity and simmetry of hand movements, presenting novel prospects for clinical applications. Indeed, exposure to artistic virtual environments olds promise or enhancing motor rehabilitation by bolstering patient motivation and treatment intensity. Virtual embodiment, characterized by the sensation of owning and controlling a virtual body, is pivotal for expanding the application of art therapy in virtual reality and the Metaverse. This approach could allow neurologic patients to partake in therapy remotely while receiving real-time monitoring from medical professionals, potentially reducing time and costs associated with hospital visits and optimizing healthcare resources.

Finally, Serino and collaborators (Serino et al., 2024) examine the role of virtual embodiment in egocentric versus allocentric spatial memory task involving bodily stimuli (pictures of hands) presented in the first-person or third-person perspectives. Their findings reveal superior memory performance in the egocentric task, irrespective of stimulus perspective. Extending these findings to the Metaverse, encounters with avatars represented in first-person or third-person perspectives offer a unique avenue for investigating object-location memory and spatial processing (Stramba-Badiale et al., 2023), presenting promising prospects for elucidating the mechanisms underlying social spatial cognition.
